# Metabolic Messengers: Extracellular Vesicles as Central Mediators of Metabolic Reprogramming in Renal Cell Cancer

**DOI:** 10.3390/biomedicines14020282

**Published:** 2026-01-27

**Authors:** Qingshu Meng, Liqun Huang, Zhiguo Chen, Rui Lin, Xiaohui Zhou, Guosheng Yang

**Affiliations:** 1 Research Center for Translational Medicine, Shanghai East Hospital, School of Medicine, Tongji University, Shanghai 200120, China; mengqingshu@alumni.tongji.edu.cn (Q.M.); lray06938@gmail.com (R.L.); 2 Department of Urology, Shanghai Geriatric Medical Center, Zhongshan Hospital Fudan University Minhang Campus, Shanghai 201104, China; bengyuyaohlq@126.com; 3 Department of Urology, Shanghai East Hospital, School of Medicine, Tongji University, Shanghai 200120, China; chenzgah@163.com

**Keywords:** renal cell carcinoma, extracellular vesicles, metabolic reprogramming, cancer, metabolism, intercellular communication

## Abstract

Renal cell carcinoma (RCC) has been described as a metabolic disease as metabolic alterations are common in disparate RCC etiologies. Extracellular vesicles (EVs), the lipid bilayer-enclosed nanoparticles secreted by all living cells, have emerged as crucial mediators of intercellular and inter-organ communication, capable of shuttling functional proteins, lipids, and nucleic acids between cells. This review summarizes the essential events in tumor-associated metabolic reprogramming with a particular focus on renal cancers. We further explore how EVs released by metabolically deranged cells in cancer with altered cargos reprogram the renal cellular landscape, fostering tumor initiation, proliferation, angiogenesis, immune evasion, and therapy resistance. Understanding this EV-mediated axis not only elucidates the pathophysiological link between these conditions but also helps to unveil novel potential therapeutic targets for RCC patients.

## 1. Introduction

Cancer remains one of the leading causes of death worldwide. Despite significant advancements in our understanding and current treatments of cancers, 90% of cancer-associated deaths are attributed to metastasis [1]. The complexity and adaptability of malignant cells constantly pose new challenges [2]. Metabolism involves a network of different biochemical reactions inside a cell that utilize nutrients to gather energy or adenosine triphosphate (ATP) and synthesize new biomolecules [3,4]. One of the fundamental hallmarks of cancer cells is their metabolism alteration which supports rapid division, resistance to cell death, and adaptation to hypoxic conditions within the tumor microenvironment (TME) [5,6].

The metabolic reprogramming of cancer cells comprises a wide range of alterations in various pathways. The phenomenon that cancer cells preferentially utilize glycolysis in the cytosol for energy production, even in sufficient oxygen, was first observed by Otto Warburg in the early 20th century, and is now defined as the Warburg effect [7]. This metabolic shift is not merely a bystander but a critical driver of tumorigenesis, aggression, and therapy resistance. Cancer cells display extensive alterations in glucose, amino acid, and lipid metabolism [8]. They use glucose for ATP production and biological macromolecule synthesis, as well as glutamine, serine, arginine, fatty acids, and lipids to facilitate their own proliferation, and seed the cancer cells in other organs, according to the concentration of local nutrients and different environments [9,10].

In addition to cancer cells, metabolic heterogeneity is also found in their surrounding cells including immune cells, fibroblasts, and mesenchymal stem cells which are important components of the tumor microenvironment [11]. Immune systems are confirmed to defend the host against cancer through distinct mechanisms. In the past few decades, immunotherapy has greatly revolutionized the cancer treatment landscape and is currently the most promising anti-tumor treatment for advanced cancers. Immune cells also undergo metabolic reprogramming during their proliferation, differentiation, and performance of their effector functions. Meanwhile, cancer metabolisms are involved in the regulation of anti-tumor immune responses via both the release of metabolites and the modulation of the expression of key immune molecules, such as lactate, prostaglandin E2, arginine, etc. [10]. Therefore, understanding metabolism abnormalities in tumors and developing metabolic interventions hold great promise for improving the effectiveness of anticancer therapies.

In this review, we summarized the essential events in tumor-associated metabolic reprogramming, evaluated the roles of extracellular vesicles (EVs) as central mediators in this process, and discussed the key obstacles remaining in unveiling the EV-mediated mechanisms of cancer progression, as well as in translating these insights into effective therapeutic strategies, with a particular focus on renal cell cancers (RCCs).

## 2. Renal Cell Cancer: A Metabolic Disease

Renal cell carcinoma is one of the most common types of urological cancer, accounting for 90% of all renal tumors. Since neither obvious clinical symptoms nor sensitive markers are available at the early stage of RCC, about 16% of the patients suffering from RCC have metastasis at the time of initial diagnosis [12]. For metastatic RCC, tyrosine kinase inhibitor (TKI) represents a first-line therapy which has shown a significant increase in overall survival (OS) and progression-free survival (PFS) [13]. However, TKI resistance remains to be a major challenge for patients with advanced RCC [14].

Recent studies suggested RCC as a metabolic disease as metabolic alterations are common in RCC [15]. For example, clear cell renal cell carcinoma (ccRCC), making up approximately 70% to 80% of diagnosed RCC [16], displays a particular histological phenotype characterized by cytoplasmic lipid deposits that underlies its pathological nomenclature [17]. Genetically, ccRCC generally reveals the inactivation of the von Hippel–Lindau (VHL) tumor suppressor gene (approximately 90% of cases), along with either mutation or promoter hypermethylation of the remaining VHL allele [15,17], which causes most of the metabolic reprogramming in RCC, such as glycolysis, tricarboxylic acid cycle (TCA) cycle, the metabolism of glutamine, ATP production, and lipid metabolism [18,19]. In the following section, we tried to briefly summarize the main metabolic anomalies in RCC and their potential therapeutic strategies.

### 2.1. Glycolysis in RCC

Glucose represents the primary source of energy in biological conditions [20]. Its homeostasis is regulated by a complex network of signals including glucose absorption, glycolysis, glycogenolysis, gluconeogenesis, glucose reabsorption, and glucose excretion [20,21]. In contrast to normal cells, in which most of the glucose is converted to pyruvate through glycolysis, cancer cells utilize lactic acid fermentation to convert pyruvate to lactate [20,21]. The ccRCC cells show enhanced glycolysis, suppressed pyruvate dehydrogenase flow, and a decreased TCA cycle compared with tumors at other sites [22]. Moreover, the rate of glycolysis in ccRCC was significantly higher than that in neighboring kidney cells [22].

In normal cells, VHL targets hypoxia-inducible factors (HIFs) for degradation. In VHL-deficient RCC cells, HIFs accumulate constitutively, which is largely responsible for the unique metabolic switch observed in ccRCC [23]. HIF signaling plays important roles in supporting the dysregulation of key glycolytic genes such as glucose transporter 1 (GLUT1), hexokinase II (HK2), and lactate dehydrogenase A (LDHA). Moreover, it selectively activates pyruvate dehydrogenase kinase (PDK) to keep glucose-derived carbons from entering the TCA cycle, increasing glycolytic activity and biomass accumulation [24,25,26]. ccRCC cells express the passive glucose transporters GLUT1. Sodium-dependent glucose co-transporter 2 (SGLT2), as the most vital member of the SGLT family, is involved in glucose reabsorption in the kidney, and the inhibition of SGLT2 by dapagliflozin reduces the glucose uptake and suppresses RCC cells growth in vivo [27]. The upregulated expression of glucose transporters such as GLUT1 and SGLT2 represents the primary phenotypic features of RCC [19]. Singer et al. found that the high expression of GLUT-1 correlates with low CD8+ T cell infiltration in tumors [28], suggesting the role of GLUT1 in the immune escape of RCC cells. Another report revealed that the increase in glucose accumulation in RCC is associated with increased resistance to TKIs [29]. PBRM1, a tumor suppressor, is the second most frequently mutated gene in cancers and the re-expression of PBRM1 in RCC cells led to decreased glucose uptake and cellular proliferation [30]. A recent study suggested that S100A2 promotes ccRCC progression through activating GLUT2 transcription, enhancing glucose uptake and amplifying glycolysis by cancer cells [31]. From a therapeutic perspective, STF-31, an inhibitor of GLUT1 was shown to selectively kill RCCs by specifically targeting glucose uptake through GLUT1 [32]. On the other hand, the inhibition of glycolysis with 2DG (the structural analog of glucose) impaired the viability and proliferation of ccRCC [33].

Renal cancer cells display a high expression of all the enzymes involved with glycolysis [34,35]. The rate-limiting gluconeogenic enzyme fructose-1,6-bisphosphatase 1 (FBP1) is known to be a tumor suppressor. Li et al., using an integrative approach comprising pan-metabolomic profiling and metabolic gene set analysis, found that FBP1 was uniformly depleted in over six hundred ccRCC samples [36]. Furthermore, FBP1 antagonized glycolytic flux, thereby inhibiting a potential Warburg’s effect and tumor growth in a ccRCC xenograft model. FBP1 also inhibited HIF transcriptional activity, while its deficiency promoted its activity [36]. A following study showed the epigenetic regulation of FBP1 in the development of renal cancer, in which the epigenetic suppression of FBP1 promoted renal tumor growth [37]. A recent study revealed that nuclear FBP1 is degraded through the ubiquitin–proteasome pathway, while the neddylation inhibitor MLN4924 could stabilize FBP1 and suppress the expressions of HIF target genes, including GLUT1, LDHA, PDK1, and vascular endothelial growth factor (VEGF), which resulted in decreased glucose uptake and lactate and NADPH production, thereby suppressing the tumor growth of ccRCC [38].

### 2.2. Lipid Metabolism in ccRCC

Lipid types mainly include phospholipids, sphingolipids, triglycerides (fats and oils), fatty acids (FAs), and sterols. They can provide energy storage, as well as act as signaling molecules and structural components of membranes, facilitating essential biological functions in the body. Lipid metabolism, as a crucial cellular process, involves the synthesis, degradation, and utilization of lipids. This metabolic reprogramming in cancers enables tumor cells to sustain their rapid growth rates, evade apoptosis, and adapt to the tumor microenvironment [39,40]. Compared to that in normal cells, the alterations of lipid metabolism in cancer cells mainly include increased de novo lipogenesis, enhanced fatty acid uptake, and altered fatty acid oxidation (FAO) [41]. Unlike many cancers that upregulate de novo lipogenesis, ccRCC is characterized by a unique duality: massive intracellular lipid and cholesterol ester storage coupled with a concurrent dependency on exogenous FAs.

Alterations in lipid metabolism are associated with worse clinical outcomes in patients with ccRCC, as lipogenic genes drive tumorigenesis. Zhang et al. found that dysregulated lipid metabolism-related gene expression in the immune microenvironment was associated with aberrant immunological activity and the reprogramming of fatty acid metabolic activity, contributing to poorer outcomes in renal cell carcinoma [42]. Recently, Simeth et al. reported that ccRCC notably accumulated lipids containing oleate which affected intratumoral CD8+ T cell infiltration and function [43]. Furthermore, they suggested a prognostic clustering of genes involved in fatty acid degradation (FAD) and cholesterol synthesis that is both ccRCC-specific and independent of major parameters such as tumor size or aggressiveness [43]. These results suggested the roles of fatty acid metabolism-based molecular signatures in the prognostic prediction and guidance of clinical drug treatment in RCC.

Metabolomics research has revealed that enzymes involved in the intrinsic FA metabolism pathway, such as ATP citrate lyase (ACLY), acetyl-CoA carboxylase (ACC), fatty acid synthase (FASN), SCD1, cluster of differentiation 36 (CD36), carnitine palmitoyltransferase 1 A, and perilipin proteins, exhibit their roles in ccRCC and each might be potential therapeutic targets [17]. ACLY, an important enzyme in FA biosynthesis that can convert citrate to acetyl-CoA, is often overexpressed in cancers and correlates with worse prognoses [44,45]. It is highly expressed in primary RCC tissues and the silencing of ACLY inhibited the proliferation and migration of RCC cells [46]. FBXW7, an E3 ubiquitin ligase involved in lipid metabolism, can interact with ACLY to promote its degradation, lower FA production, and contribute to lipid content reduction. In addition, HIF-2α/LPCAT1 contributes to the reprogramming of lipid metabolism in ccRCC by modulating the F-Box/WD Repeat-Containing Protein 7 (FBXW7)-mediated ubiquitination of ACLY [47].

ccRCC cells increase both the import and the synthesis of FAs to facilitate metabolic plasticity [48]. It was reported that an elevated CD36 mRNA expression correlates with increased visceral adipose tissue and predicts a poor prognosis in ccRCC patients [49]. A following study confirmed that hypoxia-dependent HIF-2α activation promotes lipid metabolism reprograming and the development of ccRCC via CD36 [50]. A recent study showed that CMKLR1, a G protein-coupled receptor of the protumorigenic adipokine chemerin, controlled lipid uptake through the regulation of sterol regulatory element-binding protein 1c and the CD36 scavenger receptor. The inhibition of CMKLR1 with a small molecule antagonist led to a dramatic reduction in tumor growth, lipid storage, and clear cell morphology in patient-derived xenograft models [51].

Adipokines and lipid species are reported to be potential biomarkers for diagnosis and treatment monitoring in patients with ccRCC. FA metabolism could potentially be targeted for therapeutic intervention in ccRCC as small molecule inhibitors targeting the pathway have shown promising results in preclinical models. A recent study demonstrated that GPR1 and CMKLR1, two G protein-coupled receptors of the protumorigenic adipokine chemerin, controlled lipid metabolism to support the development of ccRCC [51]. The inhibition of both receptors suppressed lipid formation and induced multiple forms of cell death [51]. Sze Kiat Tan et al. found obesity-dependent elevations of the adipokine chemerin in ccRCC tumor tissues and patient plasma [52]. They also identified that a HIF-dependent adipokine prevented fatty acid oxidation. Meanwhile the attenuation of chemerin led to a significant reduction in lipid deposition and the impairment of ccRCC growth [52]. A recent study showed that lysine acetyltransferase 2B (KAT2B) suppressed de novo lipogenesis by interfering with HDAC5-LSD1 complex assembly in RCC and the loss of KAT2B lead to elevated FASN expression, lipid accumulation, and tumor progression [53].

The dependency on lipid metabolism presents a compelling array of therapeutic vulnerabilities. For example, solute carrier family 27 member 3 (SLC27A3), which is highly expressed in lipid-rich tumors like ccRCC, is associated with a poor prognosis. The knockdown of SLC27A3 markedly suppressed LD accumulation and mitophagy and overcame pazopanib resistance in ccRCC [54]. The inhibition of SOAT1, an enzyme catalyzing the formation of fatty acid cholesterol esters, has shown remarkable preclinical efficacy in ccRCC [55]. Yin et al. suggested that ACLY mRNA or proteins are predictors of poor prognoses in ccRCC patients and that ACLY gene expression was significantly correlated with immune cell infiltration and immune inhibitors in ccRCC [56]. They further confirmed that ACLY inhibition significantly impaired cell proliferation and migration, decreased lipid droplets formation, and suppressed the epithelial–mesenchymal transition (EMT) of ccRCC [56]. Therefore, the combination of metabolic agents with immune checkpoint inhibitors is a highly promising strategy to simultaneously target both renal cancer cells and TME remodeling.

### 2.3. Amino Acid Metabolism

While the glycolytic and lipid metabolic phenotypes of ccRCC are well established, the reprogramming of amino acid metabolism is increasingly recognized as an equally critical pillar of the disease. Amino acids are not merely building blocks for proteins but key regulators of redox balance and anti-tumor immunity in renal cancer cells [57]. Previous studies indicated the rewiring of several animo acids mainly including glutamine, arginine, and tryptophan metabolism in ccRCC [19].

Glutamine, as a building block in protein synthesis and lipid synthesis, is one of the primary nutrients utilized by cancer cells [57]. It is imported into cells through its specific transporter SLC1A5 and then glutaminase (GLS) converts glutamine to glutamate and triggers glutamine catabolism [58,59]. The enzyme glutamate dehydrogenase (GDH) mediates the export of glutamate to the cytoplasm for protein synthesis or its conversion to α-ketoglutarate [58,59]. RCC cells utilize an unusual form of glutamine metabolism for lipid biosynthesis even at normal oxygen levels [60]. Reductive glutamine metabolism is reported to act as consequence of VHL loss because re-expressing functional VHL suppresses this pathway [60,61]. Compared with that in normal kidney tissues, the use of glutamine in ccRCC is increased, and the GLS/glutathione (GSH) disulfide balance is strictly regulated [60]. The upregulation of glutamine level is associated with elevated levels of free fatty acids in RCC [34,60]. In murine models of human RCC, both amounts of glutamate and α-ketoglutarate increased, along with the upregulation of GLS, which plays a vital role in glutamine metabolism, supporting the metabolic needs for rapidly proliferating cancer cells [60]. The systemic administration of GLS inhibitors can suppress the growth of RCC cells in mice xenografts [61]. CB-839, a potent and well-tolerated GLS inhibitor, shows its efficacy in patients with heavily pretreated RCC [62]. However, a recent phase I/II open-label study suggested limited anti-tumor activity with the combination of telaglenastat and nivolumab in patients with advanced/metastatic ccRCC [63].

Arginine, as a semi-essential amino acid, plays critical roles in metabolic pathways, including the biosynthesis of protein polyamines, nitric oxide, nucleotides, proline, urea, creatine, and glutamate [64]. Arginine is synthesized from citrulline (the precursor of arginine) through the urea cycle [19] and argininosuccinate synthase-1 (ASS1) acts as the rate-limiting enzyme in this process [64]. Arginine auxotrophy is a metabolic state in tumors where ASS1 activity deficiency makes the cells dependent on extracellular arginine [65]. Sciacovelli et al. found that the VHL loss-dependent reprogramming of branched-chain amino acid catabolism sustains the de novo biosynthesis of aspartate and arginine, supporting renal cancer progression [66]. A previous study found an absence or dramatic decrease in ASS1 in the biopsy samples of ccRCC patients and that those cancer cells exhibited arginine auxotrophy or a dependency on external arginine for growth [67]. The epigenetic reactivation of ASS1 render the cancer cells to acquire the capability to generate arginine, invade, and metastasize [66]. Furthermore, the deprivation of arginine suppresses tumor growth in the RCC mouse model [67]. Therefore, arginine deprivation might be an appealing approach to treat ccRCC. The enzyme arginine deaminase can catalytically deplete arginine into citrulline. ADI PEG20, a pharmacologically modified (PEGylated) variant of arginine deaminase showed promising efficacy against ccRCC tumors [67,68].

Tryptophan (TRP), an essential amino acid, is associated with three major downstream metabolic pathways including the serotonin, indoleacetate, and kynurenine (KN) pathways [19]. The KN pathway is responsible for the degradation and uptake of most dietary tryptophan [64]. The metabolism of tryptophan through the KN pathway is upregulated to generate high levels of immunosuppressants, such as kynurenine and quinolinate [19]. It is confirmed that the accumulated KN promotes the apoptosis of effector T cells and suppresses anti-tumor immune responses [69]. A recent study demonstrated that polyadenylate-binding protein, PABPC1L, can induce IDO1 to promote tryptophan metabolism and facilitate immune evasion in RCC [70].

Amino acid metabolism is not a peripheral pathway in ccRCC but is integrated into the core oncogenic program. The unique dependencies on glutamine, arginine, and TRP, coupled with the profound impact on anti-tumor immunity, make this network a rich source of therapeutic targets. Future success will depend on effectively targeting these pathways in combination with existing therapies, moving toward a multi-pronged metabolic and immunologic attack on this complex disease.

## 3. Extracellular Vesicles in RCC

Extracellular vesicles are lipid bilayer-delimited particles released by virtually all cells [71]. There are at least three main EV subtypes including exosomes, microvesicles, and apoptotic bodies, and each subpopulation differs in their biogenesis, size, and molecular cargo [72]. EVs carry biological information from their parent cells and exert their roles through the transfer of bioactive cargos including nucleic acids (such as mRNAs, non-coding RNAs, and DNA), proteins (such as signaling proteins, transcription factors, and enzymes), lipids, and metabolites [73,74,75]. The cargos of EVs depend on the origin cell types as well as the biological status of the parent cells. Previous evidence confirmed that EVs act as mediators of cell-to-cell communications [76]. For example, the transfer of EVs between cancer and immune cells facilitate tumor progression [77]. Small EVs also mediate tumor–platelet communications and activate platelets, causing thrombosis [78]. EVs can also shuttle their bioactive cargo through interstitial fluid and blood to allow inter-organ communication in both healthy and diseased states [79]. It is reported that small EVs from post-MI hearts accelerate tumor growth [80]. EVs also mediate the communication of adipose tissue with the brain to promote cognitive impairment associated with insulin resistance [81]. During hyperglycemia, liver EVs can be secreted into the circulation, and then transfer the signal to skeletal muscle and the pancreas, improving whole-body glycemic control [82]. Wang et al. recently identified tumor-derived EVs and particles (EVPs) as crucial mediators of cancer-induced hepatic metabolic reprogramming [83].

**Figure 1:**
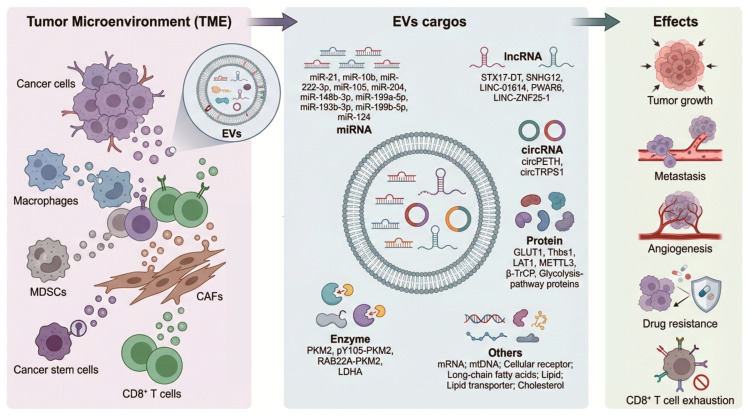
EVs derived from various cell types in the TME play crucial and diverse roles in driving tumor proliferation, invasion, immune evasion, angiogenesis, and metastasis, and inducing drug resistance by transferring distinct molecules related to metabolism.

In the TME, EVs derived from various cell types play crucial and diverse roles in driving tumor proliferation, invasion, immune evasion, angiogenesis, and metastasis by transferring oncogenic proteins, miRNAs, lncRNAs, circRNAs, enzyme, and other metabolic molecules while also inducing drug resistance (Figure 1). Increasing evidence indicated that EVs play vital roles in RCC growth and metastases via their capacity to stimulate proliferation and angiogenesis, evade detection by the immune system, and elicit resistance to treatment [84]. It is reported that C3 from RCC cell-derived EVs contributes to metastasis via fostering an immune-suppressive environment [85]. RCC-derived exosomes transfer non-coding RNAs such as lncARSR and lncRNA AP000439.2 to induce macrophage M2 polarization [86,87], or transfer circEHD2 to activate cancer-associated fibroblasts (CAFs), promoting the progression of RCC [88]. Pan et al. found that sunitinib-resistant RCC cells showed an upregulation of lncRNA IGFL2-AS1 which was associated with poor prognoses in patients with ccRCC who received sunitinib therapy. Furthermore, sunitinib-resistant RCC cell-derived EVs can transfer lncRNA IGFL2-AS1 to recipient cells, transmitting the treatment resistance [89]. Zhang et al. found that exosomes derived from RCC cells under IL-12 stimulation can induce the generation of cytotoxic T lymphocytes against RCC antigens, leading to enhanced anti-tumor effects [90]. Other studies revealed that tumor-associated macrophage-derived exosomes drive tumor cell aggression though their miRNAs cargos in RCC [91,92]. Moreover, CAFs boost the tumorigenesis and progression of RCC via exosome-mediated paracrine molecules [93,94]. Xuan et al. found that TKI-resistant RCC cell-derived exosomes had a lower expression of miR-549a than TKI-sensitive cells, which induce vascular permeability and angiogenesis to promote tumor metastasis [95]. Later studies revealed that strategies to inhibit the biogenesis of exosomes may overcome the spread of drug resistance. For instance, ketoconazole is reported to decrease tumor-specific exosomes by inhibiting the expression of Alix, nSMase, and Rab27a proteins [96]. In addition, EVs, as lipid bilayer vesicles, can effectively protect their internal RNAs from hydrolysis and thus represent promising anticancer drug carriers and tumor vaccines, which has been comprehensively summarized in a previous review [97].

## 4. Metabolic Messengers: Extracellular Vesicles as Central Mediators of RCC Metabolic Reprogramming

EVs have emerged as pivotal mediators of both intercellular and inter-organ communication, capable of systematically altering the metabolism of recipient cells within the tumor microenvironment and distant organs. On the other hand, cancer cell metabolism rewiring (e.g., the Warburg effect, glutaminolysis, increased lipid synthesis) directly influences EV biogenesis, secretion, and cargo sorting. Therefore, the relationship between EV cargo alterations and metabolic reprogramming is bidirectional and synergistic. They operate within a positive feedback loop, collectively fueling tumor progression, metastasis, and therapy resistance.

As mentioned above, the metabolic identity of ccRCC is defined by pseudohypoxia, glycolytic dependency, lipid handling defects, and amino acid auxotrophies. While the intrinsic VHL-HIF axis drives these pathways, the EV-mediated communication of this metabolic state throughout the TME is essential for RCC progression. In this section, we summarize the main findings on the role of EVs as metabolic messengers contributing to the progress of malignancy, with a special focus on renal cell cancers. Understanding this EV-mediated circuit may unveil novel therapeutic vulnerabilities in this challenging condition.

### 4.1. Dissemination of Hypoxic Signaling via EVs

Under hypoxic conditions, the biogenesis, release, and cargo loading of EVs are significantly altered. Hypoxia enhances the total secretion of EVs and, more importantly, selectively enriches them with a unique repertoire of biomolecules that mirror the aggressive hypoxic phenotype. Key components of hypoxic EVs cargo include hypoxia-regulated non-coding RNAs (mainly including miRNAs, lncRNAs, and circRNAs), proteins, and enzymes that can reprogram the metabolism of normoxic cells to a glycolytic phenotype (Table 1).

miRNAs are among the most potent and well-studied EV cargos, and can be transferred to recipient cells, silencing target mRNAs in recipient cells [98]. The roles of miRNAs in cancers have been extensively explored, revealing their dysregulation in multiple cancer types and at different stages through various mechanisms [99]. Later studies confirmed their roles in various metabolic pathways, including cholesterol and fatty acid metabolism, as well as pancreatic islet function and glucose metabolism [100]. The EV-mediated dissemination of the HIF signature via transfer miRNAs represents the most prominent mechanism in tumor progression.

miR-21 is a well-established onco-miR (cancer-promoting microRNAs) that acts as a potent positive regulator of the HIFs signaling pathway. A previous study showed that EVs-miR-21 from pulmonary epithelial cells contribute to myofibroblast differentiation via promoting glycolysis in arsenic-induced pulmonary fibrosis [101]. Hypoxic tumor-derived exosomal miR-21 promotes the metastasis of multiple cancers [102,103]. In hepatocellular carcinoma (HCC) cells, acidic conditions stimulate exosomal miR-21 and miR-10b expressions which promote HCC cell growth and metastasis [104]. In addition, tumor-associated macrophage-derived exosomal miR21-5p promotes tumor angiogenesis by upregulating Yes-associated protein (YAP1)/HIF-1α signaling in head and neck squamous cell carcinoma [105]. Another study suggested VHL as a novel direct target of miR-21. The knockdown of miR-21 increased VHL expression and thus modulated the HIF-1α/VEGF pathway in pancreatic cancer cells [106]. Moreover, the inhibition of miR-21-5p in osteosarcoma cells decreased glucose uptake, lactic acid production, and ATP level and downregulated proteins such as GLUT1, LDHA, HK2, and pyruvate kinase M2 (PKM2) [107]. Very recently, it was reported that circPETH, transported via EVs from TAMs to hepatocellular carcinoma cells, facilitates glycolysis and metastasis in recipient cells. Further results revealed that circPETH-147aa promotes PKM2-catalyzed ALDOA-S36 phosphorylation and impairs anti-HCC immunity [108].

A recent meta-analysis including 16 studies suggested that miR-210, miR-378, miR-1233, and miR-21 have a high accuracy for diagnosing RCC [109]. Growing evidence highlighted the association between miR-21 and urological tumors, especially emphasizing the impact on kidney cancers [110]. p-Cresyl sulfate (pCS), which triggers EMT, migration, and proliferation via the HIF-1α pathway, can elevate miR-21 levels, promoting cell proliferation and EMT in ccRCC cells. Blocking miR-21 resulted in reduced HIF-1α expression in pCS-treated ccRCC cells [111]. ccRCC-EVs are enriched with miRNAs that target key regulators of oxidative phosphorylation. Emerging studies suggested the role of secreted miR-210-3p as a potential biomarker for ccRCC [109,112,113,114,115]. The transfer of miR-210, a classic hypoxia-inducible miRNA, can suppress mitochondrial complex I activity and ISCU (iron–sulfur cluster scaffold homolog) in recipient cells, inhibiting oxidative metabolism and promoting a glycolytic shift [116]. Metabolome analysis revealed that miR-210 is involved in the activation of anaerobic glycolysis in the early stage of ccRCC development, helping cancer cells to acquire growth and survival advantages [117]. Meanwhile long non-coding RNA STX17-DT can be packaged into EVs, which transmit axitinib resistance to other cells in RCC by inhibiting mitochondrial reactive oxygen species (ROS) accumulation and ferroptosis [118].

Some EVs carry portions of mitochondrial DNA (mtDNA) and proteins. The transfer of mtDNA can restore respiratory function in recipient cells with defective mitochondria, aiding in chemoresistance and metastasis. mtDNA released by senescent tumor cells can be packaged within EVs and selectively transferred to polymorphonuclear myeloid-derived suppressor cells which drive immunosuppression and tumoral progression in prostate cancer through the cGAS-STING pathway [119]. An analysis of the mtDNA in serum-derived exosomes showed their relevant significance in RCC aggressiveness [120]. Tumor cells also transfer dysfunctional, ROS-generating mitochondria via EVs to T cells to induce senescence and immune evasion [121]. A recent study demonstrated that renal cancer cells uptake lactic acid via monocarboxylate transporter-1 (MCT1) when interacting with CAFs. Exosomes derived from these ccRCC cells can activate fibroblasts to trigger tumor progression through downregulating PANK3 [122].

EVs from highly glycolytic tumors contain several glycolytic enzymes including PKM2, HK2, and GLUT1. The uptake of these EVs by less aggressive cancer cells or stromal cells can enhance their glycolytic flux, promoting a Warburg-like phenotype. The exosome-mediated transfer of PKM2 from prostate cancer (PCa) cells into bone marrow stromal cells was identified as a novel mechanism through which primary tumor-derived exosomes promote premetastatic niche formation [123]. A recent study revealed a new pathway for stemness propagation and chemoresistance in non-small cell lung cancer via phosphorylated PKM2-loaded small EVs [124]. RAB22A-PKM2-rich exosomes can induce intercellular chemoresistance transmission [125]. A recent study identified a suite of sEV-associated glycolysis pathway proteins in patients with ovarian cancer which confers carboplatin resistance on target cells [126]. Morrissey et al. found that tumor-derived exosomes can induce immunosuppressive macrophages characterized by increased PD-L1 expression through glycolytic-dominant metabolic reprogramming in a pre-metastatic niche [127]. Breast cancer-derived exosomes increased the glycolysis pathway in PBMCs through the PD1-GLUT1-HK2 metabolic axis [128].

In RCC cells, TKI treatment increases both the number of large and small EVs in a dose-dependent manner. GLUT1 was enriched in sEVs which displayed increased glucose uptake and glycolytic metabolism compared with sEVs released from vehicle-treated cells [129]. This phenomenon may contribute to the development of drug resistance mechanisms in RCC. Gan et al. found that LDHA was highly expressed in ccRCC, and this high expression was associated with M2 macrophage infiltration in tumors. Moreover, the high expression of LDHA in ccRCC cells increased the EPHA2 level in tumor cell-derived exosomes and promoted the progression of ccRCC [130].

**Table 1:** Extracellular vesicle cargos and EV-mediated dissemination of hypoxic signaling.

Types	Contents	Cell Types	Target Cells	Effect	Mechanism	References
miRNA	miR-21	pulmonary epithelial cells	myofibroblasts	myofibroblast differentiation	promote glycolysis	[101]
miRNA	miR-21	hypoxic head and neck squamous cell carcinoma cells	head and neck squamous cell carcinoma	promote tumor invasion and metastasis	activation of cancer-associated fibroblasts by targeting YOD1	[102]
miRNA	miR-21	hypoxic oral squamous cell carcinoma cells	normoxic cells	educate a prometastatic phenotype	HIF-1α and HIF-2α-dependent	[103]
miRNA	miR-21/miR-10b	HCC cells in acidic microenvironment	HCC cells	HCC cell growth and metastasis	HIF-1α and HIF-2α-dependent	[104]
miRNA	miR21-5p	tumor-associated macrophage	head and neck squamous cell carcinoma	tumor angiogenesis	upregulating YAP1/HIF-1α signaling	[105]
circRNA	circPETH	TAMs	hepatocellular carcinoma cells	facilitate glycolysis and metastasis, impair anti-HCC immunity	promote PKM2-catalyzed ALDOA-S36 phosphorylation via the MEG pocket	[108]
lncRNA	STX17-DT	axitinib-resistant RCC cells	RCC	transmit axitinib resistance	inhibit mitochondrial ROS accumulation and ferroptosis	[118]
mtRNA	mtDNA	senescent tumor cells	polymorphonuclear myeloid-derived suppressor cells	drive immunosuppression and enhance chemotherapy efficacy	the cGAS-STING pathway	[119]
miRNA	miR-222-3p	RCC cells	CAFs	foster fibroblasts activation and tumor progression	downregulate PANK3	[122]
enzyme	PKM2	PCa cells	bone marrow stromal cells	promote premetastatic niche formation	HIF-1α-dependent fashion	[123]
enzyme	pY105-PKM2	cancer stem cells	non-small cell lung cancer	slower cell cycle progression, and enhanced chemoresistance	N/A	[124]
enzyme	RAB22A-PKM2	chemoresistant CRC cells	CRC cells	induce acquired drug resistance	promote the phosphorylation of synaptosome-associated protein 23	[125]
protein	glycolysis-pathway proteins	hypoxic ovarian cancer cells	normoxic ovarian cancer	platinum resistance	associated with changes in glycolysis and fatty acid synthesis	[126]
protein	GLUT1	TKI-treated RCC cells	N/A	increase secretion and glycolytic activity of sEVs	N/A	[129]
enzyme	LDHA	ccRCC	M2-type macrophages	promote the progression of ccRCC	increase the EPHA2 level which promotes M2-type polarization of macrophages by promoting activation of the PI3K/AKT/mTOR pathway	[130]

N/A indicates information that is not available.

**Figure 2:**
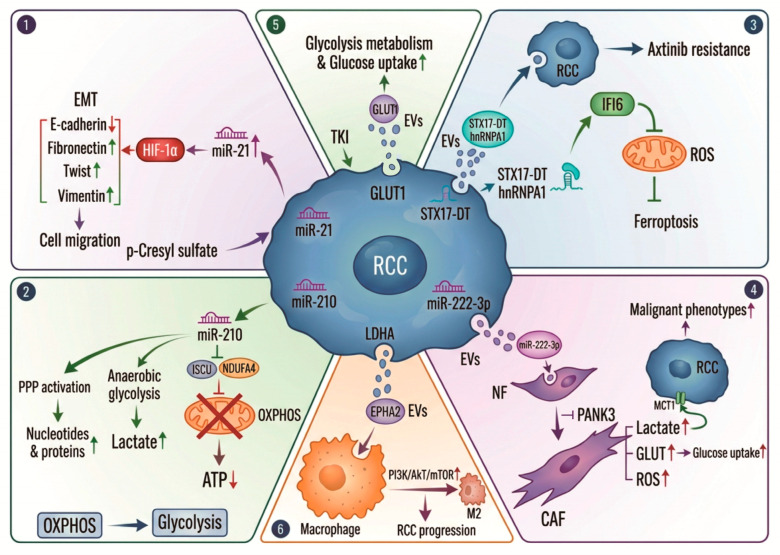
EVs enriched in non-coding RNAs, mitochondrial DNA (mtDNA), and glycolytic enzymes lead to the dissemination of hypoxic signaling, enabling communication between cells in the tumor microenvironment and contributing to the deterioration of RCC.

In summary, EVs, which are enriched in non-coding RNAs, mtDNA, and glycolytic enzymes, lead to the dissemination of hypoxic signaling, enabling communication between cells in the tumor microenvironment and contributing to the deterioration of RCC (Figure 2).

### 4.2. Transfer of Lipid-Laden Cargo and Enzymes by EVs

Increasing evidence has highlighted the role of lipid metabolism in tumorigenesis and progression. Lipids, including FAs and cholesterols, are essential components of cellular membranes, and precursors of some important signaling molecules. In addition to the membrane constituent, exosomes’ inner cargos, including enzymes, play critical roles in transducing intercellular signals in the TME [131]. Compared with their parent cells, the lipid composition of immune cell-derived exosomes displayed enrichments in sphingomyelin, gangliosides, and di-saturated lipids, and a decrease in phosphatidylcholine and diacylglycerol [132]. Meanwhile exosomes from prostate cancer cells are highly enriched in glycosphingolipids, sphingomyelin, cholesterol, and phosphatidylserine [133]. Ceramide, as one of the lipids critical for exosome formation, is enriched in this EVs subtype [134]. Lipid-enriched EVs can stimulate cell signaling pathways associated with oncogenesis and metastasis [134], and phosphatidylserine lipids have been identified as cancer detection biomarkers [76]. Table 2 shows the lipid-laden cargo and enzymes in EVs and their roles in tumor progression.

Previous studies confirmed the significant role of fatty acid receptor CD36, a primary lipid transporter, in promoting cancer progression [135,136]. The membrane localization and elevated expression of CD36 contribute to lipid uptake and downstream FAO, triggering the initiation of tumor metastasis [137]. Other studies uncovered the mechanism by which tumor cells metabolically interact with macrophages in TME via CD36 and lipid metabolism reprogramming [138,139]. Tumor cell-derived long-chain fatty acids, carried by EVs, can be preferentially partitioned into macrophages via CD36, which fuel macrophages and trigger their tumor-promoting activities [139]. In addition, EVs from ovarian cancer cells induce senescent lipid-laden macrophages to facilitate omental metastasis through CD36-dependent uptake to drive lipid accumulation [140]. Melanoma-derived EVs also regulate immunosuppressive macrophage-like characteristics and pre-metastatic niches by upregulating CD36 [141]. Susanne Pfeiler et al. found that CD36 triggered macrophage invasion and persistent tissue colonization and that this process is mediated by pancreatic tumor-derived microvesicles during metastasis [142]. A recent study revealed that the excess cholesterol derived from CRC cells was released via EVs and taken up by surrounding macrophages via CD36. Macrophages, taking up CRC cell-derived cholesterol, preferentially polarize towards M2-like TAMs, which promoted CRC malignancy [143].

**Table 2:** The lipid-laden cargo and enzymes in EVs and their roles in tumor progression.

Types	Contents	Cell Types	Target Cells	Effect	Mechanism	References
lipid	ceramide	immune cells	cancer cells	oncogenesis and metastasis	stimulate cell signaling pathways	[134]
lipid	long-chain fatty acids	liver cancer cells	metastasis-associated macrophages (MAMs)	promote cancer progression	CD36-mediated engulfing of lipid-enriched vesicles	[139]
lipid	lipid	ovarian cancer cells	senescent lipid-laden macrophages	facilitate omental metastasis	CD36-dependent uptake to drive lipid accumulation	[140]
lipid transporter	CD36	melanoma	macrophages	immunosuppressive macrophage-like characteristics and pre-metastatic niche	upregulating CD36	[141]
NA	NA	pancreatic tumor	myeloid immune cells	promotion of liver metastasis	CD36-regulated immune cell invasion	[142]
lipid	cholesterol	CRC cells	surrounding macrophages	promotion of CRC malignancy	preferentially polarize towards M2-like TAMs	[143]

N/A indicates information that is not available.

Several studies confirmed the presence of FAs and enzymes involved in lipid metabolism in EVs [144]. The lipidomic profiles of EVs from non-tumorigenic, tumorigenic, and metastatic cell lines revealed differences in the molecular lipid species, highlighting the potential significance of the EVs’ lipids in both cancer progression and as potentially useful biomarkers of diagnosis or staging [145]. FASN, a key enzyme essential for fatty acid synthesis, is upregulated in ccRCC and its expression is correlated with a poorer OS of ccRCC [146]. High sphingosine kinase (SPHK)1 expression is also detected in human ccRCC and associated with poor prognoses for ccRCC patients. The knockdown of SPHK1 inhibits ccRCC development through inhibiting the de novo synthesis of fatty acids [147]. Stearoyl-CoA desaturase (SCD) is an enzyme that controls the synthesis of unsaturated fatty acids. SCD1 expression is upregulated in ccRCC, supporting ccRCC viability [148]. High SCD increases endogenous FA desaturation, which is required for cellular proliferation when exogenous sources of monounsaturated FAs are limited [149].

Given the lipid-rich nature of ccRCC, it is possible that EVs serve as lipid carriers with functional consequences. The uptake of these lipid-rich EVs by cells like CAFs or macrophages may induce lipid accumulation, leading to either a pro-inflammatory or immunosuppressive phenotype. The current understanding about this biology is still limited and further in-depth studies are warranted.

### 4.3. Reprogramming Amino Acid Metabolism in Cancer by EVs

The reprogramming of amino acid metabolism is one of the critical hallmarks for cancers, enabling biomass production, the maintenance of redox homeostasis, and signaling activation. Recent studies unveiled a sophisticated, non-cell-autonomous EV-mediated mechanism by which tumors manipulate their microenvironment and distant sites. This section will explore the specific molecular cargo, including non-coding RNAs, proteins, and metabolites, that EVs transport to alter pathways such as glutaminolysis, mechanistic target of rapamycin complex 1 (mTORC1) signaling, and amino acid transport, thereby fostering a pro-tumorigenic niche (Table 3).

#### 4.3.1. Transfer of Non-Coding RNAs

Non-coding RNAs, as key regulators of gene expression, are abundantly packaged into EVs. They can simultaneously target multiple components of a metabolic pathway. The delivery of non-coding RNAs targeting metabolic signaling represents one of the vital mechanisms of the EV-mediated regulation of amino acid metabolism. The mTORC1 pathway is a master regulator of cell growth and is highly sensitive to amino acid availability. EVs deliver miRNAs that suppress negative regulators of mTORC1, lead to its hyperactivation and increased translation in recipient cells, even under nutrient stress. For instance, breast cancer-secreted miR-105 and miR-204 regulate amino acid-induced mTORC1 signaling and fibroblast protein synthesis during periodic nutrient fluctuations [150]. Cancer-derived exosomal miR-148b-3p induces M2 macrophage polarization via the TSC2/mTORC1 pathway to promote breast cancer cell migration and invasion [151]. Wang et al. found that miR-199a-5p carried by CAF-released EVs can induce tumor progression through regulating the FKBP5-mediated AKT1/mTORC1 signaling pathway in gastric cancer [152]. In addition to mTORC1 signaling, previous studies showed that exosomal miR-193b-3p from M2-polarized macrophages promote glutamine uptake of pancreatic cancer by targeting TRIM62 [153]. M2 macrophage-derived exosomal miR-381 attenuates urethral fibroblasts activation through inhibiting YAP/GLS1-regulated glutaminolysis [167]. Recently, Ruan et al. found that breast cancer cell-derived EVs-miR-199b-5p targets different solute carrier transporters in astrocytes and neurons to hijack their metabolic coupling, promoting brain metastasis [154]. Microglia-released sEV inhibits glioma growth by increasing the expression of glutamate transporter Glt-1 on astrocytes and enhancing glutamate clearance through transferring its cargo miR-124 [155].

Other studies showed the roles of exosomal circRNAs or lncRNAs on the rewiring of amino acid metabolism in cancers. Bladder cancer cell-derived exosomal circTRPS1 accelerates CD8+ T cell exhaustion and tumor progression in bladder cancer microenvironments via regulating GLS1-mediated glutamine metabolism [156]. The cystine/glutamate antiporter SLC7A11 is overexpressed in multiple human cancers and acts to import cystine for glutathione biosynthesis and antioxidant defense [168]. A recent study showed that lung cancer cell-derived sEVs containing lncRNA SNHG12 promote the metastasis of non-small cell lung cancer (NSCLC) via the miR-326/SLC7A11 axis [157]. Moreover, CAF-specific lncRNA LINC01614, packaged in exosomes, upregulates the glutamine transporters SLC38A2 and SLC7A5 and enhances glutamine uptake in lung adenocarcinoma [158]. In CRC, myofibroblastic CAF-derived exosomal lncRNA PWAR6 promotes CRC liver metastasis through enhancing glutamine uptake by cancer cells and depleting glutamine availability for NK cells in the tumor microenvironment [159]. Yu et al. recently reported that EV-packaged non-coding RNA linc-ZNF25-1 from pancreatic cancer cells promotes the uptake of asparagine by pancreatic stellate cells to facilitate chemoresistance [160].

#### 4.3.2. Transfer of Amino Acid Transporters, Proteins, and Metabolites

EVs can express L-type amino acid transporter 1 (LAT1) or alanine–serine–cysteine transporter 2 (ASCT2, encoded by gene Slc1a5). When fused with a recipient cell, these EVs can directly enhance their capacity to uptake specific amino acids from the environment, conferring a growth advantage. Glutamine uptake, which is mediated by ASCT2, is involved in glutathione synthesis to resolve oxidative stress [169,170,171]. A recent study demonstrated that the depletion of ASCT2 triggers oxidative stress and promotes oral carcinogenesis through the polarization of M1-like TAMs via the exosome-transferred pathway [161]. Yang et al. reported that SLC1A5 is significantly enriched in radiation-induced sEVs which promote the proliferation of unirradiated cancer cells via enhancing SLC1A5-glutamine metabolism [162]. Liu et al. suggested that amino acid transporter LAT1 expressed on cancer cell-derived exosomes may act as a diagnostic and prognostic biomarker [163]. A recent EVs-based proteomics analysis found a strong association between B7-H3, LAT1, and SLC29A1 levels and serum PSA levels and the number of bone lesions, indicating their potential as biomarkers of disease burden and therapy response [172]. Another report identified EV-derived LAT1 mRNA as a powerful inducer of the CRC aggressive phenotype [164]. In NSCLC, it is reported that CAF-derived exosomal METTL3 promoted the malignant phenotype and glutaminolysis in cancer cells by inducing amino acid transporter LAT1 (SLC7A5) m6A modification [165]. Moreover, NSCLC cell-derived exosomal β-transducin repeats-containing protein (β-TrCP) reduced amino acid transport and consequently lead to mTOR inactivation and CD8+ T cell exhaustion [166]. A recent study identified arginine as a ferroptotic promoter using a metabolites library [173]. This effect is mainly mediated by arginine’s conversion to polyamines. Moreover, ferroptotic cells produce enhanced polyamine-containing EVs into the microenvironment, and these EVs further sensitize neighboring cells to ferroptosis and accelerate the “spread” of ferroptosis in the tumor region [173].

By transferring non-coding RNAs, proteins, and metabolites, tumor or non-tumor cell-derived EVs systematically reprogram amino acid metabolism in diverse recipient cells. This process fuels tumor growth, suppresses anti-tumor immunity, and facilitates metastasis and treatment resistance. It is still unclear how EVs exert their roles in the reprogramming of amino acid metabolism in renal cancers. Understanding this EV-mediated axis may provide novel insights into the etiology of RCC.

## 5. Conclusions and Future Perspectives

Metabolic reprogramming is a central hallmark of renal cancer pathogenesis, and therapeutic strategies targeting the dysregulated metabolic pathways, such as mTOR inhibitors, 2-DG, and the glutaminase inhibitor CB-839, have emerged as a promising approach to combat this challenging malignancy [174]. Emerging studies identified that EVs are indispensable conductors of the complex metabolic orchestra within the tumor microenvironment and beyond. By transferring a diverse toolkit of miRNAs, enzymes, and metabolites, they synchronize the metabolic activities of disparate cells to support overall tumor growth and dissemination. Understanding the precise mechanisms and cargos involved is not only fundamental to cancer biology but also holds immense promise for the development of novel therapeutic strategies. However, key obstacles remain in unveiling the EV-mediated mechanisms in cancer progression, as well as in translating these insights into effective therapeutic strategies. Firstly, while the transfer of ncRNAs or proteins via EVs has been widely described, little evidence exists on the transfer of lipids or metabolites that can induce metabolic rewiring as well as trigger metastatic behaviors and/or drug resistance, especially in renal cancers. This field is open to new explorations and may lead to innovative approaches. Previous studies confirmed that both cancer cells and non-cancer cells in the TME can produce EVs which contain multiple cargos targeting diverse metabolic pathways. The metabolism–EV circuit is embedded within a web of redundant signaling pathways. Inhibiting one node (e.g., glycolysis) often leads to the compensatory upregulation of another (e.g., glutaminolysis or OXPHOS), allowing tumors to evade treatment. Moreover, EVs themselves can carry the very machinery (enzymes, miRNAs) that activate these bypass routes. In addition, technical challenges in EV targeting still exist. The research on EV biology, including its content, function, targeting, and internalization mechanisms, is in its early stages, mainly due to technical limitations in detecting, isolating, and characterizing diverse subpopulations of EVs. Investigations into the fine tailored production of EVs or the disruption of their uptake for specific cellular targets will further propel their therapeutic prospects. As tumor cell-derived EVs can regulate the metabolic rewiring of other cells within the TME, combination strategies to integrate EV-based therapies with metabolic modulators, immune checkpoint inhibitors, targeted therapies, and other emerging agents may enhance treatment efficacy and overcome RCC resistance mechanisms. Furthermore, in depth analyses of EVs from drug-sensitive and -resistant cells could help identifying the profile characterizing good or poor responses to therapy in specific tumors. Additionally, a more refined understanding of the metabolic heterogeneity within RCC subtypes, combined with advanced biomarker strategies, will lead to more personalized and effective treatments.

## Figures and Tables

**Table 3:** The specific molecular cargos that EVs transport to reprogram amino acid metabolism in cancer.

Types	Contents	Cell Types	Target Cells	Effect	Mechanism	References
miRNA	miR-105 and miR-204	breast cancer	fibroblasts	regulate stroma-produced proteins	target RAGC to regulate mTORC1 signaling	[150]
miRNA	miR-148b-3p	breast cancer cells	macrophages	promote breast cancer cell migration and invasion	induce M2 macrophage polarization via TSC2/mTORC1 pathway	[151]
miRNA	miR-199a-5p	CAF	gastric cancer cells	tumor progression	regulate FKBP5-mediated AKT1/mTORC1 signaling pathway	[152]
miRNA	miR-193b-3p	M2-polarized macrophages	pancreatic cancer	promote progression and glutamine uptake of pancreatic cancer	target TRIM62 and decrease c-Myc ubiquitination	[153]
miRNA	miR-199b-5p	breast cancer cells	astrocytes and neurons	brain metastases	target different solute carrier transporters to hijack their metabolic coupling	[154]
miRNA	miR-124	microglia	astrocytes	inhibit glioma growth	increase the expression of Glt-1 on astrocytes and enhance glutamate clearance	[155]
circRNA	circTRPS1	bladder cancer cells	CD8+ T cells	accelerate CD8+ T cell exhaustion and tumor progression	regulate GLS1-mediated glutamine metabolism	[156]
lncRNA	lncRNA SNHG12	lung cancer cells	TAMs	promote the metastasis of NSCLC	miR-326/SLC7A11 axis	[157]
lncRNA	lncRNA LINC01614	CAFs	lung adenocarcinoma	promote cancer progression	upregulate the glutamine transporters SLC38A2 and SLC7A5	[158]
lncRNA	lncRNA PWAR6	myofibroblastic CAFs	CRC	promote CRC liver metastasis	enhance glutamine uptake by cancer cells and deplete glutamine availability for NK cells	[159]
lncRNA	linc-ZNF25-1	pancreatic cancer cells	pancreatic stellate cells	facilitate chemoresistance	uptake of asparagine	[160]
protein	Thbs1	oral carcinogenesis	M1-like TAMs	promote oral carcinogenesis	oxidative stress triggered by ASCT2 deletion promotes oral carcinogenesis through Thbs1-mediated M1 polarization	[161]
cellular receptor	SLC1A5	radiated cancer cells	unirradiated cancer cells	promote the proliferation of unirradiated cancer cells	enhance glutamine metabolism	[162]
protein	amino acid transporter LAT1	pancreatic, biliary tract, and ovarian cancer cells	N/A	act as a diagnostic and prognostic biomarker	N/A	[163]
mRAN	LAT1	CRC	different cancer cell lines	powerful inducer of CRC aggressive phenotype	N/A	[164]
protein	METTL3	CAFs	non-small cell lung cancer	promote the malignant phenotype and glutaminolysis	induce amino acid transporter LAT1 (SLC7A5) m6A modification	[165]
protein	β-TrCP	NSCLC cells	CD8+ T cells	mTOR inactivation and CD8+ T cell exhaustion	induce YAP1 ubiquitination and CD8+ T cell exhaustion	[166]

N/A indicates information that is not available.

## Data Availability

No new data were created or analyzed in this study. Data sharing is not applicable to this article.

## Abbreviations

The following abbreviations are used in this manuscript:

ACC	Acetyl-CoA Carboxylase
ACLY	ATP Citrate lyase
ASCT2	Alanine–Serine–Cysteine Transporter 2
ASS1	Argininosuccinate Synthase-1
ATP	Adenosine Triphosphate
β-TrCP	β-transducin repeats-containing Protein
CAFs	Cancer-associated Fibroblasts
ccRCC	Clear cell Renal Cell Carcinoma
CD36	Cluster of Differentiation 36
CPT1A	Carnitine Palmitoyltransferase 1A
CRC	Colorectal Cancer
EMT	Epithelial–Mesenchymal Transition
EVs	Extracellular Vesicles
FAD	Fatty Acid Degradation
FAO	Fatty Acid Oxidation
FAs	Fatty Acids
FASN	Fatty Acid Synthase
FAT1	FAT atypical cadherin 1
FBP1	Fructose-1,6-bisphosphatase 1
FBXW7	F-Box/WD Repeat-Containing Protein 7
GDH	Glutamate Dehydrogenase
GLS	Glutaminase
GLUT1	Glucose Transporter 1
HCC	Hepatocellular Carcinoma
HIFs	Hypoxia-Inducible Factors
HK2	Hexokinase II
KAT2B	Lysine Acetyltransferase 2B
KN	Kynurenine
LAT1	L-type Amino Acid Transporter 1
LDHA	Lactate Dehydrogenase A
MCT1	Monocarboxylate transporter-1
mtDNA mTORC1	Mitochondrial DNA mechanistic Target of Rapamycin Complex 1
NSCLC	Non-small Cell Lung Cancer
OS	Overall Survival
PCa	Prostate cancer
pCS	p-Cresyl Sulfate
PDK	Pyruvate Dehydrogenase Kinase
PFS	Progression-free Survival
PKM2	Pyruvate Kinase M2
RCC	Renal Cell Carcinoma
ROS	Reactive Oxygen Species
SCD	Stearoyl-CoA desaturase
SGLT2	Sodium-dependent Glucose co-Transporter 2
SLC27A3	Solute Carrier family 27 member 3
SPHK	Sphingosine Kinase
TCA	tricarboxylic acid cycle
TKI	Tyrosine Kinase Inhibitor
TME	Tumor Microenvironment
TRP	Tryptophan
VEGF	Vascular Endothelial Growth Factor
VHL	Von Hippel–Lindau
YAP1	Yes-associated Protein
